# Cannabis abuse and brain morphology in schizophrenia: a review of the available evidence

**DOI:** 10.1007/s00406-012-0346-3

**Published:** 2012-08-21

**Authors:** Berend Malchow, Alkomiet Hasan, Paolo Fusar-Poli, Andrea Schmitt, Peter Falkai, Thomas Wobrock

**Affiliations:** 1 Department of Psychiatry and Psychotherapy, University Medical Center Göttingen, Georg-August-University, von-Siebold-Strasse 5, 37075 Göttingen, Germany; 2 Department of Psychosis Studies, Institute of Psychiatry, King’s College London, London, UK; 3 Department of Psychiatry and Psychotherapy, Ludwig Maximilian University, Munich, Germany; 4 Center of Mental Health, County Hospitals Darmstadt-Dieburg, Gross-Umstadt, Germany

**Keywords:** Cannabis, Substance abuse, Magnetic resonance imaging, Schizophrenia, High-risk subjects, First-episode patients

## Abstract

Substance abuse is the most prevalent comorbid psychiatric condition associated with schizophrenia, and cannabis is the illicit drug most often abused. Apart from worsening the course of schizophrenia, frequent cannabis use especially at an early age seems to be an important risk factor for developing schizophrenia. Although a large body of neuroimaging studies gives evidence for structural alterations in many different brain regions in schizophrenia patients, there is still limited knowledge of the impact of cannabis abuse on brain structure in schizophrenia. We performed a systematic review including structural magnetic resonance imaging studies comparing high-risk and schizophrenia patients with and without cannabis abuse and found inconclusive results. While there is some evidence that chronic cannabis abuse could alter brain morphology in schizophrenia in patients continuing their cannabis consumption, there is no convincing evidence that this alteration takes place before the onset of schizophrenia when looking at first-episode patients. There is some weak evidence that cannabis abuse could affect brain structures in high-risk subjects, but replication of these studies is needed.

## Introduction

Substance abuse in individuals with schizophrenia is very common; it has become the most prevalent comorbid psychiatric condition associated with schizophrenia and contributes to an unfavourable disease course [65]. Apart from the legal substances such as tobacco and alcohol, cannabis is the most illicit drug abused in schizophrenia patients. Depending on the study, the comorbidity rates are within a range from approximately 15 to 65 % [8, 37]. In a population-based study, the prevalence of substance use disorder in patients with schizophrenia was estimated to be 4.6 times higher than in the general population [46]. Substance abuse, especially cannabis, has also been discussed as an important risk factor for developing schizophrenia [34]. At least substance abuse may contribute to an earlier onset of schizophrenia as seen in many first-episode studies [6].

Persisting comorbid substance abuse is associated with negative outcome in schizophrenia, including more frequent and longer periods of hospitalization, higher relapse rates even in first-episode patients, elevated rates of extrapyramidal motor symptoms (EPS), lower medication compliance, higher rates of unemployment, violence, higher rate of criminality and an increased risk of committing suicide [10, 23, 28, 31, 45, 49, 54, 56]. Hypotheses explaining the possible aetiological relationship between the two disorders describe concepts of common increased vulnerability to both disorders with regard to dysfunctional affect regulation or deficits with stress coping, of secondary substance abuse (in the context of self-medication or increased sensitivity to drug effects), of secondary psychotic disorder triggered or induced by substance use and of a bidirectional maintenance of both disorders by neurobiological or psychological interaction [16, 36]. The effects of abused substances on schizophrenia symptoms vary, making it difficult to differentiate between substance abuse–related symptoms and those related to functional psychosis [16]. For example, alcoholic hallucinosis shares some features with paranoid schizophrenia, and the differentiation of both diseases may be difficult [55].

Differences in psychopathology between schizophrenia patients with and without substance abuse were reported inconsistently in the recent literature. Comorbid substance abuse has been associated with higher positive symptomatology [6, 9, 53], lower negative symptoms [4, 39, 53], fewer positive and negative symptoms [13] and no significant differences in psychopathology at admission and follow-up [67].

Brain abnormalities identified in schizophrenia using neuroimaging techniques reveal evidence for structural and functional alterations in multiple brain regions focussing on frontal cortex, temporal cortex, thalamus, hippocampal complex, basal ganglia and even cerebellum [1]. Meta-analyses and reviews of published studies concerning volumetric measurements obtained by magnetic resonance imaging have demonstrated ventricular enlargement and volume decrease especially of frontal and temporal lobe structures in schizophrenia [14, 18, 50, 68]. A meta-analysis of volumetric structural MRI studies investigating the hippocampus reported a 4 % bilateral volume reduction in schizophrenic patients [38], while some studies observed pronounced decrease in hippocampal structures only on the left side [51]. Abnormalities of temporal lobe structures linked to mesolimbic system were suggested as responsible for cognitive and emotional disturbance commonly seen in schizophrenia, and temporal volume reductions have been linked to clinical features. Especially reduced bilateral hippocampal size has been associated with memory deficits, and the decrease in total volume of superior temporal gyrus (STG) correlates with the severity of thought disorder and auditory hallucinations [3, 51].

In first-episode and antipsychotic-naïve patients, results of volumetric MRI studies are inconclusive [59]. Ventricular enlargement in antipsychotic-naive patients up to 20 % compared to healthy controls has been observed in most studies, but volume changes of cerebral structures are less conclusive. While most studies failed to detect a volume reduction in basal ganglia [59], a decreased thalamus volume was frequently reported (in the range of 5 –18 % of thalamus volume compared to healthy controls) [15, 19, 20]. Additionally, grey matter of frontal and temporal lobe structures, in particular of the entorhinal cortex and the hippocampus, was found to be reduced [21, 27, 57].

There is still limited knowledge about the influence of substance abuse on brain morphology in schizophrenia patients. In summary, the unfavourable effect of cannabis abuse on the course of schizophrenia and the link between cerebral volume loss and poor outcome in schizophrenia, and a negative effect of substance abuse on brain volumes in schizophrenia patients can be hypothesized. This review focusses on the question whether comorbid patients do show more structural cerebral changes than patients without ongoing or previous cannabis abuse or not.

## Methods

### Search strategy, data sources and study selection

To achieve a comprehensive overview of the available neuroimaging studies dealing with our topic (structural volumetric measurement by MRI in schizophrenia patients with comorbid cannabis abuse), a systematic literature search and study evaluation were performed according to the Cochrane-Criteria, using the computerized search databases PubMed (1966–2012) and the Web Of Knowledge. Only studies published in English language were considered for this review. The keywords used were “schizophrenia” or “psychosis”, “sMRI” or “structural imaging” and “cannabis” or “marihuana” or “marijuana” or “tetrahydrocannabinol”. The search was limited to “humans”. The literature obtained from the search was carefully screened, based on the title and abstract by two independent researchers. Only the appropriate articles were selected, their full text was reviewed, and the study outcomes were critically evaluated. The reference lists of these articles were checked for relevant studies on the field that were overlooked by the initial literature search.

## Results

We found 105 hits in the databases, and after evaluating the abstracts, we could include 16 studies covering our topic in the review. The studies with their results are summarized in the Tables 1, 2 and 3.

**Table 1:** Overview of structural magnetic resonance imaging studies in first-episode (FES) or early-onset (EOS) schizophrenia and comorbid cannabis abuse

Study	Methods	Design	Participants	Findings
Cahn et al. [7]	sMRI, 1.5 Tesla, 1.2 mm thickness; ROI; VBM	Cross-sectional	First-episode schizophrenia with (*N* = 27) and without (*N* = 20) cannabis abuse	No difference in volumes of total white or grey matter, cerebellum and caudate; reduced asymmetry of the lateral ventricles in the SZ-SUD-subgroup
Szesko et al. [58]	sMRI, 1.5 Tesla, 1.5 mm thickness; ROI	Cross-sectional	First-episode schizophrenia with (*N* = 20) and without (*N* = 31) cannabis abuse and healthy controls (*N* = 56)	Cannabis abuse is associated with less anterior cingulate grey matter; no grey matter volume loss in frontal temporal gyrus or orbitofrontal gyrus
Bangalore et al. [2]	sMRI, 1.5 Tesla, 1.5 mm thickness; VBM, a priori single mask consisting of hypothesized CB1-rich regions (bilaterally) that included DLPFC, hippocampus, posterior cingulate and cerebellum	Cross-sectional	First-episode schizophrenia with (*N* = 15) and without (*N* = 24) abuse and healthy controls (*N* = 42)	Decrease in grey matter density in the right posterior cingulate cortex (PCC) in SZ-SUD
Rais et al. [43]	sMRI, 1.5 Tesla, 1.2 mm thickness; VBM	Cross-sectional and Longitudinal	First-episode schizophrenia with (*N* = 19) and without (*N* = 32) Cannabis abuse and healthy controls (*N* = 31)	No morphological difference between the two groups at the beginning of the study at five-year follow-up SZ-SUD shows more pronounced brain volume reduction and larger increase in lateral and third ventricle volumes than SZ-NSUD
Peters et al. [40]	sMRI, DTI	Cross-sectional	Male ROS (*N* = 35) with and without a history of cannabis use before the age of 17, matched HC (*N* = 17)	ROS with cannabis use before the age of 17 showed increased directional coherence in the bilateral uncinate fasciculus, anterior internal capsule and frontal white matter. These abnormalities were absent in FOS without cannabis use before the age of 17
Wobrock et al. [66]	sMRI, 1.5 Tesla, 1.0 mm thickness; ROI	Cross-sectional	First-episode schizophrenia (recent-onset psychosis) with (*N* = 20) and without (*N* = 21) substance abuse (essentially Cannabis abuse)	No differences in the assessed temporolimbic brain morphology (superior temporal gyrus (STG), amygdala–hippocampus complex and cingulum) between the two subgroups
Dekker et al. [12]	sMRI, DTI	Cross-sectional	EOS with regular use of cannabis before the age of 15 (*N* = 10), EOS with regular use of cannabis at the age of 17 years or later (*N* = 8), EOS who were cannabis naïve (*N* = 8), HC (*N* = 10)	Cannabis-naïve EOS showed reduced white matter density and reduced fractional anisotropy in the splenium of the corpus callosum compared with EOS with early-onset cannabis use. In the same brain area, cannabis-naïve EOS showed reduced fractional anisotropy compared with HC
Rais et al. [44]	sMRI, 1.5 Tesla, 1.2 mm thickness	Cross-sectional, cortical thickness measurement	ROS (*N* = 51) and HC (*N* = 31), 5-year follow-up: *N* = 19 ROS with cannabis abuse, *N* = 32 without cannabis abuse	Thinning of right supplementary motor cortex, inferior frontal cortex, superior temporal gyrus, angular gyrus, occipital and parietal lobe compared to controls, compared to ROS without cannabis abuse thinning of left DLPFC, left ACC and left occipital lobe demonstrated
Cohen et al. [11]	sMRI, 1.5 Tesla, 1 mm thickness	Cross-sectional	First-episode schizophrenia with (*N* = 6) and without (*N* = 13) Cannabis abuse and healthy controls (*N* = 19) and healthy cannabis abusers (*N* = 17)	FES displayed lower total cerebellar grey matter than HC, no difference between FES with and without cannabis abuse in adolescent and young adults
James et al. [26]	sMRI, DTI, VBM	Cross-sectional	EOS with (*N* = 16) and without (*N* = 16) early cannabis abuse and HC (*N* = 28)	EOS with cannabis abuse showed GM density loss in temporal fusiform gyrus, parahippocampal gyrus, ventral striatum, right middle temporal gyrus, insular cortex, precuneus, right paracingulate gyrus, dorsolateral prefrontal cortex, left postcentral gyrus, lateral occipital cortex and cerebellum. Similar group comparison showed decreased fractional anisotropy (FA) in particular in brain stem, internal capsule, corona radiata, superior and inferior longitudinal fasciculus in EOS with cannabis abuse
Kumra et al. [29]	sMRI, ROI	Cross-sectional	Adolescents with EOS (*N* = 35), cannabis abuse (*N* = 16), EOS + cannabis abuse (*N* = 13), and healthy controls (HC) (*n* = 51)	A significant EOS-by-CUD interaction was observed. In the left superior parietal region, both “pure” EOS and “pure” CUD had smaller grey matter volumes that were associated with lower surface area compared with HC. A similar alteration was observed in the comorbid group compared with HC, but there was no additive volumetric deficit found in the comorbid group compared with the separate groups. In the left thalamus, the comorbid group had smaller grey matter volumes compared with the CUD and HC groups

*sMRI* structural magnetic resonance imaging, *DTI* diffusion tensor imaging, *ROI* region of interest, *VBM* voxel-based morphometry, *GM* grey matter, *WM* white matter, *SUD* substance use disorder (substance abuse or dependency), *NSUD* no substance use disorder (no substance abuse or dependency), *SZ* schizophrenia, *FES* first-episode schizophrenia, *EOS* early-onset schizophrenia, *HC* healthy controls

**Table 2:** Overview of structural magnetic resonance imaging studies in high-risk or prodromal schizophrenia patients with comorbid cannabis abuse

Study	Methods	Design	Participants	Findings
Habets et al. [22]	sMRI, cortical thickness	Cross-sectional	Patients with schizophrenia (*N* = 88), healthy siblings at higher than average genetic risk for schizophrenia (*N* = 98), healthy controls (*N* = 87)	Patient group displayed reductions in cortical thickness for cannabis exposure, a similar pattern was found in the sibling–control comparison for cannabis
Welch et al. [61]	sMRI, 1 Tesla, ROI;	Longitudinal	Subjects at high risk for developing schizophrenia exposed (*N* = 25) and not exposed (*N* = 32) to cannabis in the intervening period of two years	Cannabis exposure was associated with bilateral thalamic volume loss over time
Welch et al. [62]	sMRI, 1 Tesla, ROI;	Cross-sectional	Subjects at high risk for developing schizophrenia (*N* = 147) and healthy controls (*N* = 36) cannabis abuse	Level of cannabis use correlated significantly and positively with volume increase in the left and right lateral ventricles and third ventricle

*sMRI* structural magnetic resonance imaging, *DTI* diffusion tensor imaging, *ROI* region of interest, *VBM* voxel-based morphometry, *GM* grey matter, *WM* white matter, *SUD* substance use disorder (substance abuse or dependency), *NSUD* no substance use disorder (no substance abuse or dependency), *SZ* schizophrenia, *FES* first-episode schizophrenia, *EOS* early-onset schizophrenia, *HC* healthy controls

**Table 3:** Overview of structural magnetic resonance imaging studies in schizophrenia patients (not FES or EOS) with comorbid cannabis abuse

Study	Methods	Design	Participants	Findings
Solowij et al. [52]	sMRI, 3 Tesla, ROI, semi-automated methods	Cross-sectional	Patients with schizophrenia (*N* = 17, 47 % long-term heavy cannabis users), healthy controls (*N* = 31, 47 % long-term heavy cannabis users)	Cerebellar white matter volume was reduced in cannabis users with and without schizophrenia compared to healthy non-users, by 29.7 % and 23.9 %, respectively, and by 17.7 % in patients without cannabis use. Healthy cannabis users did not differ in white matter volume from either of the schizophrenia groups. There were no group differences in cerebellar grey matter or total volumes. Total cerebellar volume decreased as a function of duration of cannabis use in the healthy users
Ho et al. [24]	sMRI, 3 Tesla, slice thickness 3 mm, ROI; CNR1-genotyping;	Cross-sectional	Schizophrenia patients without (*N* = 183) and with (*N* = 52) cannabis abuse	Patients with cannabis abuse/dependence had smaller fronto-temporal WM volumes than patients without heavy cannabis use. Significant genotype-by-cannabis use interaction effects on WM volumes

*sMRI* structural magnetic resonance imaging, *DTI* diffusion tensor imaging, *ROI* region of interest, *VBM* voxel-based morphometry, *GM* grey matter, *WM* white matter, *SUD* substance use disorder (substance abuse or dependency), *NSUD* no substance use disorder (no substance abuse or dependency), *SZ* schizophrenia, *FES* first-episode schizophrenia, *EOS* early-onset schizophrenia, *HC* healthy controls

### Volumetric sMRI studies in schizophrenia (not first-episode) with comorbid cannabis abuse

Surprisingly, we could not identify MRI studies including patients with chronic schizophrenia or a disease course with multiple episodes and solely cannabis abuse.

A MRI study including 17 patients with schizophrenia (mean illness duration, above 2 years) and 31 healthy controls with 48 % of the healthy group and 47 % of the patients being long-term heavy cannabis users was performed focussing on the cerebellum [52]. In cannabis users with and without schizophrenia, cerebellar white matter volume was reduced compared to healthy non-users, by 29.7 and 23.9 %, respectively, and by 17.7 % in patients without cannabis use. Healthy cannabis users did not differ in white matter volume from either of the schizophrenia groups. No group differences in cerebellar grey matter or total volumes could be detected. Interestingly, total cerebellar volume decreased as a function of duration of cannabis use in the healthy users, and psychotic symptoms as well as illness duration correlated with cerebellar measures differentially between patients with and without cannabis use. The authors conclude that long-term heavy cannabis use in healthy individuals is associated with smaller cerebellar white matter volume similar to that observed in schizophrenia, but volume reduction is even more pronounced in patients with schizophrenia using cannabis.

In a further study including 235 schizophrenia patients (14 % with comorbid cannabis abuse, 8 % with cannabis dependence), patients with marijuana abuse/dependence had smaller fronto-temporal white matter (WM) volumes than those without heavy marijuana use [25]. Genotyping for cannabinoid receptor 1 (CB1/CNR1) demonstrated significant CNR1 (rs12720071) genotype-by-marijuana-use interaction effects on WM volumes and neurocognitive impairment. Furthermore, CNR1 genetic polymorphisms were associated with WM brain volume variation among schizophrenia patients. These findings suggest that heavy cannabis use in the context of specific CNR1 genotypes may contribute to greater WM volume deficits and cognitive impairment.

### Volumetric sMRI studies in recent-onset or first-episode schizophrenia with comorbid cannabis abuse

Our literature research revealed that most published studies investigated first-episode or recent-onset schizophrenia patients in comparison with healthy subjects. However, the results of these neuroimaging studies are again heterogeneous and remain inconclusive.

In one study, patients with recent-onset schizophrenia (schizoaffective disorder) with comorbid substance abuse (*n* = 20, all THC, 7 patients with additional amphetamines/ecstasy consumption, 2 with opiates, 8 with cocaine, 1 with hallucinogens and 2 with alcohol) did not differ significantly in terms of volumetric morphometry in temporolimbic regions (superior temporal gyrus, amygdala–hippocampus complex, cingulum) from schizophrenia patients without previous substance abuse (*n* = 21) [66]. However, this study did not include a healthy control group. In a further study of this research group, another sample suffering from first-episode schizophrenia (*n* = 47) and healthy controls (*n* = 55) underwent MRI scanning, and volumes of hippocampus, superior temporal gyrus, prefrontal cortex, internal capsule (ALIC) and the cross-sectional areas of the corpus callosum were compared between subgroups of patients with (*n* = 19) (all cannabis, 4 with additional amphetamines/cocaine) and without previous substance abuse (*n* = 28). A significant reduction in the cross-sectional area of the C 2-segment of the corpus callosum in the non-abusing patients in comparison with the patients with previous cannabis abuse could be observed, suggesting more subtle brain abnormalities in the group without previous substance abuse [32].

Another study investigating brain volumes in cannabis-exposed patients with first-episode schizophrenia compared to non-exposed patients revealed no differences between the subgroups for total brain volume, total grey and white matter, ventricles, cerebellum and caudate except a decreased asymmetry of the lateral ventricles in the cannabis-exposed patients [7]. In a recent longitudinal study, the same research group could demonstrate that first-episode schizophrenia patients with continued use of cannabis (*n* = 19) showed increased loss of cerebral grey matter volume and larger increases in lateral and third ventricle volumes than patients who did not use cannabis during the follow-up (*n* = 32) [43]. Nevertheless, brain volumes of schizophrenia patients with and without cannabis abuse did not differ significantly at the beginning of the longitudinal study, although it has to be assumed that the patients abused cannabis several years before entering the study. The same group of researchers also performed an analysis of cortical thickness in these patient groups [44]. At the beginning of the investigation, cortical thickness did not differ between patients and controls and between cannabis-using and non-using patients. During the follow-up period, excessive thinning of the right supplementary motor cortex, inferior frontal cortex, superior temporal gyrus, angular gyrus, occipital and parietal lobe in patients relative to controls after controlling for the factor “cannabis use” was observed. Patients who used cannabis showed additional thinning in the left dorsolateral prefrontal cortex (DLPFC), left anterior cingulate cortex (ACC) and left occipital lobe as compared to those not using cannabis during the observation interval.

A further study investigating the volumes of the superior frontal gyrus, anterior cingulated gyrus and orbital frontal lobe found less anterior cingulate grey matter in cannabis-abusing first-episode schizophrenia patients compared with patients with no history of cannabis abuse and healthy volunteers [58]. The association between reduced volume in anterior cingulate gyrus (ACC) and the history of cannabis abuse in schizophrenia has been discussed in the context of a disturbed function of ACC with the consequence of poor decision-making and more compulsive drive towards drug use as a predisposition to substance abuse and not as a toxic effect of cannabis itself. This is in contrast to one study revealing no volumetric differences in the ACC, the amygdala–hippocampus complex, the STG and the lateral ventricles between recent-onset schizophrenia patients with and without previous cannabis abuse [66]. In this study, the patients without previous cannabis abuse tended to demonstrate more reversed temporal asymmetry as the non-users. This may be a subtle hint that the subgroup of cannabis abusers lower their threshold of psychosis through drug abuse itself and the other subgroup increases the vulnerability by presenting slightly more brain abnormalities.

In a more recent voxel-based morphometry (VBM) study, a decrease in grey matter density in the right posterior cingulate cortex (PCC) in the subgroup of first-episode schizophrenia with cannabis abuse was observed compared to those patients without cannabis abuse [2]. The other included brain regions with high density of CB1 receptors, for instance hippocampus, did not differ between these groups. The authors concluded that cannabis use may be associated with altered brain structure in particular regions (in their study PCC) with a high density of CB1 receptors.

A magnetic resonance imaging study focussing on cerebellar grey and white matter in first-episode patients with and without a history of cannabis use and non-psychiatric cannabis users revealed a lifetime dose-dependent regional reduction in grey matter in the right cerebellar lobules and a tendency for more profound grey matter reduction in lobule III with younger age at the onset of cannabis use [11]. First-episode patients had lower total cerebellar grey matter/total cerebellar volume ratio and marked grey matter loss in the vermis, pedunculi, flocculi and lobules compared to pairwise matched healthy controls. This pattern and degree of grey matter loss did not differ from age-matched first-episode patients with comorbid cannabis use, indicating only small dose-dependent effects of juvenile cannabis use on cerebellar neuropathology but no evidence of an additional effect of cannabis use on cerebellar grey matter pathology in psychosis.

In an adolescent MRI study, subjects with early-onset schizophrenia (EOS, *n* = 35), cannabis use disorder (CUD, *n* = 16), comorbid EOS with CUD (*n* = 13) and healthy controls (HC) (*n* = 51) were compared with regard to brain volume within frontal, temporal, parietal and subcortical ROIs [29]. If volumetric differences in one ROI were identified, cortical thickness and surface area of this ROI were additionally measured and compared between subgroups. The authors found a significant EOS-by-CUD interaction, in the left superior parietal region; both “pure” EOS and “pure” CUD had smaller grey matter volumes that were associated with lower surface area compared with HC. A similar alteration was observed in the comorbid group compared with HC, but there was no additive volumetric deficit in the comorbid group compared with the separate groups (EOS and CUD).

In a diffusion tensor imaging (DTI) study, 35 male recent-onset schizophrenia patients, with and without a history of cannabis use before the age of 17, and 21 matched healthy comparison men without illicit drug use were assessed [40]. In comparison with healthy controls, patients with cannabis use before the age of 17 showed increased directional coherence in the bilateral uncinate fasciculus, anterior internal capsule and frontal WM. These abnormalities were absent in patients without cannabis use before the age of 17, but not related to lifetime doses of cannabis or other illicit drugs. The authors concluded that recent-onset schizophrenia patients starting to use cannabis during early adolescence may represent a subgroup of schizophrenia patients with increased WM directional coherence reflecting structural hyperconnectivity. However, they contrast their results with the fact that in most DTI studies, schizophrenia patients were associated with hypoconnectivity [48].

Another study investigated male schizophrenia in early adolescence patients with regard to abnormalities in WM structure and integrity using high-resolution structural and diffusion tensor brain imaging and compared patients who started a regular use of cannabis (1) before the age of 15 years (early-onset cannabis users, *n* = 10) or (2) at the age of 17 years or later (late-onset cannabis users, *n* = 8), (3) cannabis-naïve patients (*n* = 8) and a healthy control group (*n* = 10) [12]. Cannabis-naïve patients showed reduced WM density and reduced fractional anisotropy in the splenium of the corpus callosum compared with patients with early-onset cannabis use. In the same brain area, cannabis-naïve patients showed reduced fractional anisotropy compared with healthy controls, indicating alterations in fibre density, degree of myelination and WM integrity. The authors suggest that the age of onset of cannabis use is not an identifying characteristic for WM abnormalities in schizophrenia patients and that cannabis-naïve schizophrenia patients may have a more vulnerable brain structure which may responsible for the onset of psychosis.

In a further VBM and DTI study comparing patients with adolescent-onset schizophrenia (AOS) with early cannabis use (more than 3 times/week for at least 6 months) (*n* = 16) to AOS without cannabis use (*n* = 16) and healthy adolescents (*n* = 28), those with cannabis abuse showed increased GM density loss in temporal fusiform gyrus, parahippocampal gyrus, ventral striatum, right middle temporal gyrus, insular cortex, precuneus, right paracingulate gyrus, dorsolateral prefrontal cortex, left postcentral gyrus, lateral occipital cortex and cerebellum [26]. In addition, cannabis-abusing AOS demonstrated decreased fractional anisotropy (FA) in particular in brain stem, internal capsule, corona radiata, superior and inferior longitudinal fasciculus compared to those without cannabis abuse. Interestingly, there were no cognitive differences between patients with and without cannabis abuse, but both subgroups impaired relative to controls.

### Volumetric sMRI studies in high-risk or prodromal schizophrenia patients with comorbid cannabis abuse

Our search revealed three studies accessing cortical volumes in high-risk subjects and pointing towards an effect of cannabis in this population. In the Edinburgh high-risk study (EHRS), individuals at high familial risk of schizophrenia were scanned at the point of entry into the study and approximately 2 years later. This study focussed on volumetric measurements of the thalamus and the amygdala–hippocampus complex. Compared to high-risk individuals not exposed to cannabis (*n* = 32), subjects with cannabis consumption (*n* = 25) revealed a bilateral thalamic volume loss over time [62].

A study investigating cortical thickness in several brain regions in schizophrenia patients (*n* = 88), their healthy siblings (*n* = 98) and healthy control subjects (*n* = 87) showed a significant interaction of cortical thickness with cannabis use in the patient and the sibling groups, but not in the healthy control group. This finding displays stronger reductions in cortical thickness in subjects with cannabis use in the patient and genetic high-risk group [22]. These results allowed the authors to hypothesize that gene–environment interactions may underlie this type of brain alteration observed in patients with schizophrenia and their relatives.

In another study including 147 at-risk subjects and 36 healthy controls, a dose-dependent increase in ventricular volume was associated with alcohol and cannabis use [61]. Additionally, alcohol and cannabis abuse were associated with an increased subsequent risk of schizophrenia. This may provide evidence that the use of cannabis or alcohol by people at high genetic risk of schizophrenia is associated with brain abnormalities (ventricular volume increase) and later risk of psychosis.

## Discussion

We reviewed structural MRI studies in schizophrenia patients and at-risk subjects with comorbid cannabis abuse to elucidate the interplay between cannabis consumption, schizophrenia and volumetric brain alterations. The results of the identified neuroimaging studies are heterogeneous and inconclusive, which may be due to several reasons.

First, we should keep in mind that the heterogeneity of the definition of the regional volume (ROIs) boundaries, differences in MRI techniques and volume extractions in previous studies make it difficult to compare volumetric measurements. Apart from selection bias of study samples, there seems to be a systematically difference when comparing volume data using VBM or the region of interest (ROI) method.

Secondly, selection of study samples may have an enormous influence on the reported results taking the mainly small numbers of patients and healthy subjects into account. For instance, decreases in grey and WM volume over time may be related to the duration, type and cumulative dose of antipsychotic medication patients received [24].

Most studies are cross-sectional and include recent-onset psychosis or first-episode schizophrenia patients, comparing those with and without comorbid cannabis abuse at a certain time point in their disease course. To evaluate the impact of comorbid cannabis abuse over time and to make a statement about a possible neurodegenerative or even toxic process, a within-subject comparison in a longitudinal design (e.g., prodromal state, first-episode state, recent-onset state, chronic state) would be an applicable way. Another important issue is the impact of antipsychotic medication and of the disease course. Although one could assume that the influence of antipsychotic medication in first-episode patients is not as strong as in chronic patients, other parameters like the duration of illness (DUI) and the duration of psychosis (DUP) may have a significant influence on brain volume more relevant than previous cannabis abuse.

From this point of view, the longitudinal studies of Rais et al. [43] are of certain interest. While finding no significant differences at study entry between subgroups, the authors could demonstrate an increased loss of cerebral grey matter, a larger increase in ventricle volume and an increased thinning of cortical thickness in the left DLPFC, left ACC and left occipital lobe in those first-episode patients who continued their cannabis abuse. This implies an additional influence of cannabis on brain structures apart from schizophrenia itself. A very interesting work analysed brain volumes of 211 schizophrenia patients in a longitudinal study design with a long follow-up period (follow-up scan was performed after 2, 5 and 9 years and every 3 years thereafter) [24]. The authors showed that antipsychotic treatment was associated with the strongest reduction in grey matter volumes, whereas illness severity had only little effect [24]. With regard to this review and our hypothesis, it is surprising that alcohol and illicit drug misuse (including cannabis abuse) had only an effect on lateral ventricles and on cerebellar volumes, but not on total brain tissue and hippocampal structures. However, after correction for other variables (antipsychotic medication, illness duration, illness severity) alcohol/illicit drug misuse, the effects did not remain significant [24]. Therefore, the main finding of this largest longitudinal study investigating brain volumes in schizophrenia patients is that the relationship between antipsychotic treatment and decrease in brain volumes in schizophrenia patients over time remains significant, even after correction for alcohol/illicit drug misuse and for illness severity/duration [24], [30]. Many studies include comorbid patients consuming other substances than cannabis, for example, amphetamines, cocaine and sometimes alcohol, making it difficult to focus on the effect of cannabis alone. Although the numbers of these patients are small, an influence of the additional consumed drugs could not be definitely excluded, especially elevated alcohol consumption is known to reduce grey matter volume [33].

Some studies of recent-onset psychosis patients include subjects with schizoaffective disorder, which may also influence volumetric measurement because these patients show a different disease course, may display minor cognitive dysfunction and have a shorter DUI than schizophrenia patients [66].

The MR studies of high-risk subjects suggest there is an additional effect of cannabis use on brain structure (e.g., reduction in thalamic volume) in this highly vulnerable population for the development of later schizophrenia. But the number of the available studies (*n* = 3) is too small to allow further conclusions, and the results need to be replicated.

### Influence of cannabis on brain structure

When discussing the influence of cannabis on brain structure in schizophrenia patients, it is important to have a look on the influence of cannabis on brain structure itself, shortly summarizing the results of neuroimaging studies in cannabis abusers without schizophrenia. Therefore, it is noteworthy that the structural neuroimaging studies in cannabis abusers without schizophrenia revealed conflicting results [42]. For instance, while two studies could not demonstrate an influence of cannabis on brain morphology, especially on hippocampus, compared to controls [5, 60], a third study found lower grey matter volumes in the right parahippocampal region, lower WM density in the left parietal region as well as increased volumes of bilateral fusiform gyrus, right thalamus and left parahippocampal region in the group of chronic cannabis users [35]. Other investigators detected only less frontal white matter volume percentage in the group of substance abusers (cannabis, opiates and cocaine) compared to healthy controls [47]. Another study without healthy control group revealed a significant correlation between age of first use and decreased total brain volume [63]. Subjects who started using marijuana before the age of 17, compared to those who started later, revealed smaller whole brain and per cent cortical grey matter and larger per cent WM volumes [63].

Although no data regarding the amount of cannabis consumption were given in most studies, one could speculate whether differences in cannabis use are responsible for the conflicting results. Most studies did not differentiate between different types of cannabis or the average amount of cannabis per use. The content of psychoactive tetrahydrocannabinol varies widely across different types of cannabis [41]. This important limitation complicates the exploration of dose–response relationships in the cited studies.

## Conclusions

In summary, there is some evidence that chronic cannabis abuse could alter brain morphology in schizophrenia in patients continuing their cannabis consumption, but there is no convincing evidence that this alteration takes place before the onset of schizophrenia when looking at first-episode patients. There is some weak evidence that cannabis abuse could affect brain structures in high-risk subjects, but replication of these findings is needed. However, even if previous cannabis substance abuse seems not to lead to pronounced structural brain abnormalities in schizophrenia [66], it may cause functional changes on cortical inhibition processes and synaptic transmission involving mainly the GABAergic, glutamatergic and dopaminergic system in schizophrenia patients and unaffected cannabis consumers [64]. This effect of cannabis on distinct brain functions including regulation of cortical excitability [17] could be the reason why cannabis consumption is associated with a higher risk of developing schizophrenia and not primarily pronounced structural brain alterations. It is conceivable that this disinhibition following cannabis abuse might lead to a consecutive neurotoxicity leading to subtle alterations in brain structures. Well-designed studies and the combination of brain imaging and neurophysiological techniques will open the doors to new possibilities for the investigation of these important questions.
